# The role of caspase-8 in the tumor microenvironment of ovarian cancer

**DOI:** 10.1007/s10555-020-09935-1

**Published:** 2020-10-07

**Authors:** Izabela Kostova, Ranadip Mandal, Sven Becker, Klaus Strebhardt

**Affiliations:** 1 Department of Gynecology, University Hospital, Goethe-University, Theodor-Stern-Kai 7, 60590 Frankfurt am Main, Germany; 2 German Cancer Research Center (DKFZ), Im Neuenheimer Feld 280, 69120 Heidelberg, Germany; 3 German Cancer Consortium (DKTK) partner site, Frankfurt, Germany

**Keywords:** Tumor microenvironment, Caspase-8, Non-apoptotic functions, Macrophages, TAMs, Ovarian cancer treatment

## Abstract

Caspase-8 is an aspartate-specific cysteine protease, which is best known for its apoptotic functions. Caspase-8 is placed at central nodes of multiple signal pathways, regulating not only the cell cycle but also the invasive and metastatic cell behavior, the immune cell homeostasis and cytokine production, which are the two major components of the tumor microenvironment (TME). Ovarian cancer often has dysregulated caspase-8 expression, leading to imbalance between its apoptotic and non-apoptotic functions within the tumor and the surrounding milieu. The downregulation of caspase-8 in ovarian cancer seems to be linked to high aggressiveness with chronic inflammation, immunoediting, and immune resistance. Caspase-8 plays therefore an essential role not only in the primary tumor cells but also in the TME by regulating the immune response, B and T lymphocyte activation, and macrophage differentiation and polarization. The switch between M1 and M2 macrophages is possibly associated with changes in the caspase-8 expression. In this review, we are discussing the non-apoptotic functions of caspase-8, highlighting this protein as a modulator of the immune response and the cytokine composition in the TME. Considering the low survival rate among ovarian cancer patients, it is urgently necessary to develop new therapeutic strategies to optimize the response to the standard treatment. The TME is highly heterogenous and provides a variety of opportunities for new drug targets. Given the variety of roles of caspase-8 in the TME, we should focus on this protein in the development of new therapeutic strategies against the TME of ovarian cancer.

## Ovarian cancer

Ovarian cancer is the fifth most common cause of death among female cancer patients and the most lethal malignancy of the female reproductive tract [1]. The poor disease outcome is primarily due to the lack of appropriate methods for early detection, increasing chemoresistance and limited surgical debulking [2]. More than 75% of the patients are already at an advanced stage of the disease at the time of diagnosis. Despite the surgical removal of the tumor and aggressive chemotherapy, most patients experience recurrence within the next 16 to 22 months. As a result, ovarian cancer has a 5-year survival rate of only 46% [3].

Histologically, ovarian cancer is divided into four subgroups: serous, endometroid, mucinous, and clear cell [4]. Serous ovarian carcinomas (SOC) are the most common epithelial carcinomas. SOC are subdivided into high grade (HGSOC) and low grade (LGSOC). Eighty-five to ninety percent of all SOC has been classified as high grade. HGSOC is associated with very high mortality and occurs mainly in elderly patients. The pathogenicity of the disease is also influenced by epigenetic and genetic alterations, represented by 10–15% BRCA mutations and 60–80% mutations or loss of TP53. LGSOC are less common, representing 2% of all ovarian carcinomas. They affect women at a young median age and have a 10-year survival rate of about 50%. Younger patients usually develop endometroid carcinoma or clear cell carcinoma, which are associated with endometriosis. Clear cell carcinoma is very rare, but it has the worst prognosis of all ovarian carcinomas with high resistance to platinum-based therapy. In contrast, endometroid carcinoma is associated with better disease outcome [4, 5].

Only 2–3% of the ovarian carcinomas are identified as mucinous carcinomas. Seventy-five percent of the cases show KRAS mutations and 20% HER2 amplifications. Usually, mucinous carcinomas can be diagnosed at an early stage and have a very good prognosis after surgical removal. [4, 5].

Tothill et al. had clustered HGSOC, LGSOC, and endometroid carcinoma into six molecular subtypes (C1–C6) using gene expression profiling [6]. High-grade tumors are clustered mainly in C1, C2, C4, and C5, while C3 and C6 are likely to be low grade. The C1 subtype has the worst prognosis and shows enhanced expression of stromal genes, desmoplasia (growth of fibrotic tissue), and metastases. C2 is the cluster with the highest immune signature, associated with high T cell activation and infiltration. The C3 subtype is characterized by low malignant potential (LMP). These tumors show enhanced expressions and mutations of the mitogen-activated protein kinase pathway genes KRAS and BRAF. C4 has a differentiated signature. Similar to C2, C4 also has high infiltration of immune cells. Upregulation of genes expressed in the mesenchymal development has been observed in the C5 subtype, which is also associated with bad disease outcome. Tumors from the C6 subtype are characterized as low-grade endometroid with overexpression of transcriptional targets of the beta-catenin/LEF/TCF complex [6]. The immunological C2 and differentiated C4 subtypes, characterized by their high infiltration of immune cells and better prognosis, also have the highest expression of cytoplasmic caspase-8 and NF-κB [7]. The co-expression of caspase-8 and NF-κB in these tumors suggests an important functional interplay between both proteins, leading to an active immune response and best overall survival (OS). In contrast, the mesenchymal subtype C5, which has a bad OS, is associated with the lowest expression of both caspase-8 and NF-κB [7]. Obviously, ovarian cancer is a highly heterogeneous disease and their precise classification into subgroups is important for the development of suitable therapies for each subtype.

The standard therapy includes surgical debulking, followed by chemotherapy or rarely radiation. The most frequently used anti-cancer drugs are platinum compounds, inducing DNA damage; taxanes, targeting the microtubule polymerization; doxorubicin, which inhibits topoisomerase II; and gemcitabine, a nucleoside analog, which induces irreparable errors after incorporation into the DNA [3]. Advanced treatments are currently based mainly on targeted therapies, which include compounds against a specific marker, involved in oncogenic mechanisms or chemoresistance, e.g., olaparib, a PARP inhibitor, which prevents DNA repair, or bevacizumab, a monoclonal antibody against VEGF-A with antiangiogenic effects [8]. Immunotherapies, boosting the immune system, are still less effective in the treatment of ovarian cancer, and there is not yet any immunotherapy for ovarian cancer approved by the FDA. The response to hormone therapies has been shown to be moderate. They can be mainly used in endometrial cancers, expressing the estrogen receptor [3].

Overall, the current therapies are not sufficient to overcome advanced ovarian cancer and most of the patients experience disease recurrence (25% of early-stage and 80% of advanced-stage patients), chemoresistance (90% of the patients in advanced stage), or high toxicity. It is therefore highly necessary to search for new therapeutic strategies to improve the overall survival of the patients. One of these is to target the components of the tumor microenvironment (TME) [3, 8]. In the last few years, the TME has been recognized to be a crucial factor in tumor development, progression, and even response to anti-cancer therapy. A better understanding of the complex interplay between the tumor and the TME could elicit new therapies and diagnostic markers, enabling the early estimation of risk from cancer or therapy resistance.

Furthermore, the microenvironment in ovarian cancer has been shown to alter the protein expression and cell signaling in the tumor cells, supporting their invasiveness and suppressing apoptosis and immune response [9]. One of the main proteins, involved in cell cycle, apoptosis, invasive and metastatic behaviors, immune cell homeostasis, and cytokine production is caspase-8 [10]. Gynecological cancers such as breast and ovarian cancers seem to be more aggressive when caspase-8 is downregulated [7, 11]. Dysregulated caspase-8 expression causes an imbalance between the apoptotic and non-apoptotic functions not only in the primary tumor but also in the TME. Caspase-8 may therefore be the link in the crosstalk between the tumor and the TME.

## Apoptotic and non-apoptotic functions of caspase-8

**Fig. 1:**
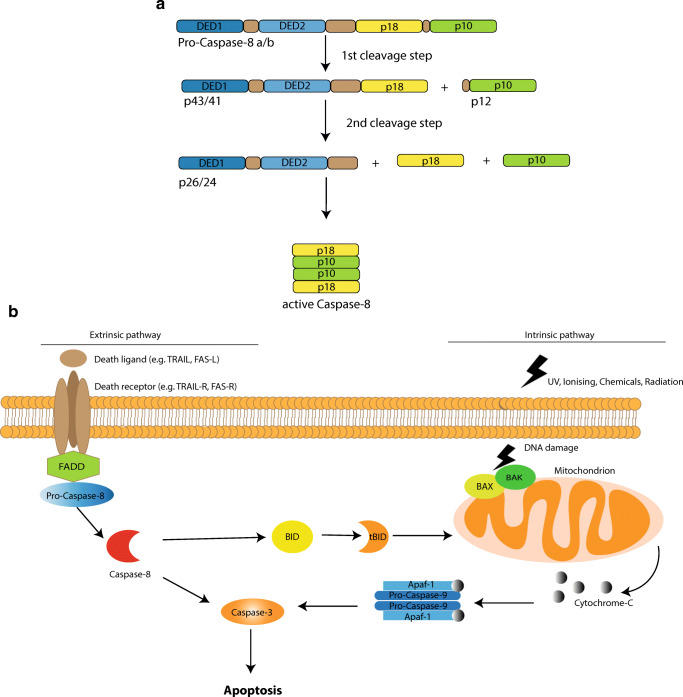
**a** Structure of pro-caspase-8 and cleavage to active caspase-8. **b** Extrinsic and intrinsic apoptotic pathways: the extrinsic signal pathway starts with the stimulation of a member of the TNF-receptor superfamily, e.g., Fas receptor (CD95/APO-1), TRAIL-R1 and R2 (DR4 and DR5), DR3 or DR6, and the assembly of the death-inducing signaling complex (DISC). Two isoforms of pro-caspase-8 (pro-caspase-8a and -8b) are involved in the formation of the DISC. After their proteolytic processing, the mature caspase-8 dissociates from the DISC and translocates to the cytosol, where it initiates apoptosis by targeting substrates such as Bid (B cell lymphoma 2 (BCL-2) homology domain 3 (BH3) only protein) or effector caspases [17, 18]. The cleavage of Bid by caspase-8 amplifies the apoptotic signal by activating the intrinsic apoptotic pathway. Cleaved Bid migrates from the cytosol to the outer mitochondrial membrane and interacts with Bax and Bak. This complex allows release of cytochrome c and the activation of Apaf1/caspase 9 apoptosome [19]

Caspases are aspartate-specific cysteine proteases with essential roles in apoptosis (caspase-2, -3, -6, -7, -8, -9, -10) and immune response (caspase-1, -4, -5, -8, -12) (Fig. 1) [12]. The apoptotic caspases are classified into two major groups: initiator and effector/executor caspases. Initiator caspases (caspase-2, -8, -9, -10) get activated by their recruitment to multiprotein complexes upon death-inducing or inflammatory signals or DNA damage response. The activation of the effector caspases (caspase-3, -6, -7) requires their proteolytic cleavage by mature initiator caspases, leading to the subsequent cleavage of downstream pro-apoptotic molecules [13]. Caspase-8 plays a crucial role in the extrinsic apoptotic pathway after external stimulation of the death receptors, leading to rapid cell death. There are two apoptosis-associated isoforms of caspase-8: pro-caspase-8a and pro-caspase-8b. Both of them are composed of a prodomain, consisting of two death effector domains (DED1 and DED2) and catalytic domain, built by the subunits p18 and p10 and a linker between them. Pro-caspase-8a differs from pro-caspase-8b by a longer linker between the prodomain and catalytic domain (Fig. 1a) [14]. The activation of pro-caspase-8a/b occurs after the formation of the death-inducing signaling complex (DISC). The first cleavage generates two subunits: p43/41, consisting of DED1, DED2, and p18, and the p12, containing p10 and the linker. The second cleavage step forms p26/24, p18, and p10. Finally, p18 and p10 assemble to form a heterodimer: p18_2_-p10_2_ [14]. The programmed cell death, mediated by caspase-8, is regulated by several mechanisms [15]. The extrinsic apoptotic pathway can be blocked after activation of caspase-8 by X-linked inhibitor of apoptosis protein (XIAP). It cleaves the effector caspases-3 and -7 downstream of caspase-8. Further regulators of apoptosis are the FLICE-like inhibitory protein (cFLIP) family. cFLIP long (cFLIP_L_) is a pro-caspase-8-like protein without catalytic activity, which is also part of the DISC and plays a dual role in this signal pathway. Low levels of cFLIP_L_ enhance apoptosis, whereas high levels inhibit caspase-8. In contrast, cFLIP short (cFLIP_S_) blocks the recruitment of caspase-8 to the DISC and prevents the oligomerization of p18 and p10 [14, 15].

Cells in which caspase-8 alone is sufficient to activate pro-caspase-3 in response to apoptotic stimuli are classified as type I cells. In type II cells, caspase-8 requires the additional activation of the intrinsic apoptotic pathway to induce cell death. In this case, caspase-8 enables a crosstalk between the extrinsic and intrinsic apoptotic pathways by cleaving BID to truncated BID (tBID) which in turn activates the intrinsic apoptotic pathway and amplifies the death-inducing signal (Fig. 1b) [16].

Besides its classical apoptosis-inducing function, caspase-8 has also many non-apoptotic and non-enzymatic functions in autophagy, anoikis, pyroptosis, inhibition of necroptosis, invasion, metastases, embryonic development, NFκB activation, immune cell homeostasis, inflammatory response, and cytokine release [10, 14]. The fact that depending on the cancer type, caspase-8 is upregulated, downregulated, unaffected or post-translationally altered, suggests that its pro- and non-apoptotic functions are decided in a tumor entity-specific manner. In normal ovaries, caspase-8 activation has been observed during the late luteal phase [20]. During this phase, prostaglandin F_2_-alpha (PGF_2_-alphaα) acts on the corpus luteum, inducing the reduction of progesterone levels and luteolysis (structural and functional degradation of the corpus luteum). At the 18-h time-point after the PGF_2_-alpha stimulation, there is an increase in the expressions of Fas receptors on the corpus luteum and FasL, as well as the activation of caspase-8. Up to 18 weeks post conception, caspase-8 has been found to be upregulated in the ovaries (www.ebi.ac.uk /gxa/home). During this period, apoptosis occurs in a highly specific manner to guarantee the normal development of the fetus [21].

The highest number of genetic alterations of caspase-8 has been registered in head and neck, uterine, cervical, and gastric cancers (www.cbioportal.org) involving somatic, frameshift, and missense mutations [22–24]. The loss of caspase-8 expression occurs very frequently in neuroendocrine cancers such as neuroblastoma, medulloblastoma, and glioblastoma [25]. In ovarian cancer, the genetic modifications of caspase-8 are estimated at 2.4%. The majority of them are amplifications of the CASP8 gene (www.cbioportal.org). Braga et al. evaluated the expression levels and epigenetic alterations of caspase-8 in normal ovarian tissues, ovarian serous cystadenoma tumors, and epithelial ovarian cancer [26]. The study found 25 times higher expression levels of caspase-8 in metastatic cancers (50.8) as compared to ovarian serous cystadenoma (2.0) and primary EOC (2.16). The CpG island methylation in the caspase-8 promoter was found in 11.8% of normal tissues and 20% of cystadenoma. All of the primary EOCs were unmethylated. When comparing primary and metastatic EOCs, hemimethylation was detected in 20% of the metastatic EOCs. The significant differences in the expression and methylation of caspase-8 in the metastatic EOCs as compared to the primary tumor and normal tissue could be associated with its non-apoptotic functions leading to apoptosis resistance, dysregulated proliferation, and enhanced activation of NF-κB, PKB/Akt, and MAPKs. However, the promotor DNA methylation was not associated with the expression levels of caspase-8, suggesting additional mechanisms, regulating the gene expression [26]. Furthermore, RNA-seq analysis had confirmed the upregulated caspase-8 in ovarian adenocarcinoma, when compared to normal ovaries (www.ebi.ac.uk/gxa/home). However, the expression of caspase-8 in the ovaries has been found to be lower in recurrent tumors, as compared to primary tumors (www.ebi.ac.uk/gxa/home).

The investigation of genetic and epigenetic alterations of caspase-8 in cancers provides better understanding of their impact on the therapy response. Cancers with low caspase-8 expression or mutations, which block its pro-apoptotic activity, may not respond to the standard treatments, which usually rely on apoptosis induction [27]. Such cancers could be targeted through necroptosis, because the absence of caspase-8 or its enzymatic activity prevents the cleavage of RIPK1 and stabilizes the necrosome [14, 27]. It is still unclear how exactly caspase-8 is involved in chemoresistance in ovarian cancer. A xenograft ovarian cancer mouse model demonstrated that the autophagy inhibitor, chloroquine, promoted the accumulation of p62 and increased caspase-8 levels, which improved the response to cisplatin and enhanced apoptosis [28]. The same group showed decreased caspase-8 activation and platin resistance in p62-mutant ovarian cancer cells. Caspase-8 and p62 have been therefore proposed as prognostic biomarkers and oncotargets for individualized therapies [28]. Moreover, two research groups had independently described the essential role of caspase-8 in the first-line therapy of ovarian cancer. The cell line CP70 was fivefold resistant to cisplatin, as compared to A2780. CP70 harbored reduced caspase-8 protein level. The combination of cisplatin with rhTRAIL significantly increased apoptosis as compared to cisplatin or rhTRAIL monotherapy [29]. The same effect has been demonstrated in the SKOV-3 and TOV-21G cell lines by Braga et al. [30]. Caspase-8 seems to be a good prediction marker for therapy response, which can be used to predict chemoresistance.

Drug-induced caspase-8 expression could be an attractive therapeutic opportunity in tumors in which the downregulation of caspase-8 leads to tumor progression, immunoescape, and increased secretion of immunosuppressive and tumor-supportive proteins into the TME. The restoration of the expression of caspase-8 has been shown in breast cancer, neuroblastoma, and medulloblastoma using decitabine and azacytidine, nucleoside analogs, which promotes the demethylation of caspase-8 promotor [31]. Furthermore, azacytidine has been found to reduce the immunosuppressive TME in an ovarian cancer mouse model. Through type I INF signaling, azacytidine provokes the recruitment of anti-tumoricidal immune cells and enhances the efficacy of immune checkpoint inhibitors [32]. The expression of caspase-8 could also be upregulated by INF-*γ*, a type II INF, acting on interferon-sensitive response elements, located within the caspase-8 promotor and mediating its transcriptional activity [31]. In addition, INF-*γ* favors the development of the anti-tumorigenic M1 macrophages and cytotoxic T cells. Further opportunities to induce the expression of caspase-8 include retinoic acid through upregulation of phospho-CREB [31]. Proteosomal inhibitors, such as bortezomib and NPI-0052, can also elevate the total cellular levels of caspase-8 through blocking its degradation [25].

In many tumors with poor prognosis, such as hepatocellular carcinoma [33], cervical cancer [34], and melanoma [35], caspase-8 was found in high levels within the nucleus. Müller et al. had investigated the nuclear expression of caspase-8 in melanoma cells and identified a hitherto unreported nuclear localization signal (NLS) (_21_SLKFLSLDY_29_) and nuclear export signal (NES) (_468_FTLRKKLVF_476_), at the N- and C-terminus of caspase-8, respectively, which enable its shuttling between the nucleus and cytosol. Only after processing of pro-caspase-8 into its active form and the removal of the NES-containing N-terminus, caspase-8 remains in the nucleus, proving that the nuclear localization of caspase-8 is possible [35].

The presence or absence of caspase-8 determines the invasive and migration behavior of the cancer cells. In breast and ovarian cancers, the downregulation of caspase-8 seems to be associated with bad prognosis and therapy resistance [7, 11].

## The tumor microenvironment

The TME consists of circulating cancer cells, non-cancerous cells (e.g., fibroblasts, macrophages, lymphocytes, and adipocytes), a variety of soluble factors (cytokines, chemokines, growth factors), extracellular matrix (ECM), blood, and lymphoid vessels. During tumor progression, the TME is constantly modulated in order to support tumor growth, angiogenesis, metastases, immune escape, and inflammation. The TME is highly heterogeneous and dynamic, not only during the different cancer stages but also at different locations: within the tumor (locally), around the tumor (regional), and in other organs (distant, metastatic). A complex crosstalk between the primary tumor and cells in other organs facilitates a favorable microenvironment for circulating cancer cells, leading to their extravasation and colonization. Such interactions have been postulated between breast cancer cells and bone cells [36] or between ovarian cancer cells and omental adipocytes [37], favoring these tissues as preferred locations for metastasizing cells. The altered cytokine production in tumor-associated stromal cells in breast cancer is also associated with epigenetic changes, leading to a role of the primary tumor as a modulator of the gene expression in neighboring cells [38, 39]. The immune response undergoes modifications as well, e.g., the co-culture between cancer cells and immune cells was shown to induce a switch into an immunosuppressive or tumor-supportive phenotype [40, 41].

The TME is, however, not only created and modulated by the tumor, it transforms from a passive participant in tumorigenesis to an active player in tumor progression [39]. Even after removing the primary tumor, the TME is able to activate the accumulating dormant cancer cells, cancer cells in a quiescent state, leading to metastases years or decades later. The TME is therefore suggested to be a crucial factor in the switch between dormancy and metastatic growth [42].

In ovarian cancer, ascites, a buildup of fluid in the abdomen, represents the major TME, providing favorable conditions for tumor growth, metastases, chemoresistance, inhibition of the immune response, and shorter overall survival. The development of malignant ascites is one of the most significant hallmarks of the disease. It is present in more than one third of the newly diagnosed patients and in almost all cases of recurrence [43].

Ovarian cancer cells detach from the primary tumor as single cells or cell aggregates, called spheroids. Before leaving the primary tumor site, they often undergo epithelial-to-mesenchymal transition (EMT), which eases the metastasizing process. Unlike most of the tumors, which spread in the body through the vasculature, ovarian cancer cells disseminate within the peritoneal cavity through a passive mechanism, carried by the physiological movement of the peritoneal fluid. Upon arriving at the metastasizing sites, preferentially the omentum and the peritoneum, the tumor cells revert to an epithelial phenotype and colonize. The intra-abdominal spread of the cancer cells increases the production of the peritoneal fluid due to the leakiness of the tumor vascularity, obstruction of the lymphatic vessels, and secretions of the tumor, resident stromal and immune cells [43, 44].

The TME, therefore, provides many different components, which can possibly be targeted by the different therapeutic strategies. Current clinical trials are testing the efficacy and toxicity of the treatments against the different targets within the TME such as pro-angiogenic factors; cytokines and chemokines and their receptors; and inhibitory receptors on cancer cells, which prevent the immune response. Immunotherapies, including CD4^+^ lymphocytes, NK cells, and autologous monocytes, are also of current interest to modulate the immunosuppressive TME in ovarian cancer (Table 1) [2, 8, 45].

**Table 1:** Clinical trials. The table represents some examples for antibodies and immunotherapies, tested in ovarian cancer patients to target specifically the TME

Drug name	Mechanism of action	Trials
Bevacizumab	Monoclonal antibody against vascular endothelial growth factor (VEGF)	Phase 2: in recurrent ovarian cancer patients (NCT01305213)
Siltuximab	Antibody against IL-6	Phase 1/2: in patients with solid tumors, including ovarian cancer (NCT00841191)
Tocilizumab	Antibody against IL-6 receptor	Phase 1: combination with chemotherapy in recurrent epithelial ovarian cancer (NCT01637532)
Nivolumab	Anti-PD-1 antibody	Phase 1: combination with WT1 vaccine in recurrent ovarian cancer (NCT02737787)
Nivolomab and Ipilimab	Anti-PD-1 antibody and CTLA-4 antibody	Phase 2: combination of immune check point inhibitors in ovarian cancer, breast cancer and gastric cancer (NCT03342417)
Pembrolizumab	Anti-PD-1 antibody	Phase 2: Pembrolizumab following weekly Paclitaxel treatment for Platinum-resistant ovarian, fallopian tube or peritoneal cancer (NCT03430700)
Pembrolizumab with Gemcitabin and Cisplatin	Anti-PD-1 antibody	Phase 2: in recurrent Platinum-resistant ovarian cancer (NCT02608684)
NK immunotherapy	Natural Killer (NK) cells	Phase1/2: combination with cryotherapy in recurrent ovarian cancer (NCT02849353)
V3-OVA vaccine	Immunotherapy with a vaccine containing ovarian cancer antigens	Phase 2: in ovarian cancer patients (NCT03556566)
Autologous monocytes	Immunotherapy	Phase 1: combination with Sylatron(R) (Peginterferon alfa-2b) and Actimmune(R) (Interferon gamma-1b) in ovarian cancer (NCT02948426)
Plerixafor	Immunostimulant to mobilize hematopoietic stem cells	Phase 1: in high-grade serous ovarian, advanced pancreatic and colorectal adenocarcinomas (NCT03277209)
Rintatolimod with pembrolizumab and cisplatin	Rintatolimod: immunomodulatory double strained RNA Pembrolizumab: anti-PD-1 antibody	Phase 1/2: in recurrent ovarian cancer (NCT03734692)
Tremelimumab with Oplaparib	Anti-CTLA-4 antibody and PARP inhibitor	Phase 2: in recurrent ovarian, fallopian tube or peritoneal cancer (NCT04034927)

## Tumor-associated macrophages

More than 50% of the cells in the peritoneal TME in ovarian cancer are comprised of tumor-associated macrophages (TAM) [46], promoting cancer-related inflammation, tumor growth, and immunosuppression. Targeting the TAMs, therefore, provides an attractive opportunity to modulate the TME. TAMs originate from two main sources: (1) the bone marrow, producing circulating monocytes, and (2) the embryonic yolk sac, producing tissue-resident macrophages. Under normal conditions, circulating monocytes in the bloodstream are recruited by different cytokines and chemokines to the inflammation site, where they differentiate into macrophages and accomplish their functions like antigen presentation, phagocytosis, activation, and recruitment of additional immune cells. Tissue-resident macrophages are localized within tissues with high proclivity toward invasion and accumulation of foreign material such as bacterial and viral particles (e.g., liver, lymph nodes, and lung). They have a long half-life, ranging from several months to years and regulate the tissue-specific immune response [46, 47].

Monocytes are modulated by the tumor in response to soluble factors in the surrounding milieu. Two major phenotypic groups of differentiated macrophages are likely to be found in the TME: anti-tumorigenic M1 or pro-tumorigenic M2 macrophages. In general, IFNγ and IL-12 induce the polarization of macrophages into the M1 phenotype, while IL-4, IL-10, IL-6, and CCL2 stimulate the M2 phenotype. Macrophages are phenotypically heterogeneous and plastic, meaning that M1 macrophages can switch to M2 and vice versa. The distinction between M1 and M2 is drawn between the expression of surface markers such as CD86, TLR2, TLR4, and MHC II for M1 macrophages and CD163, CD206, and CCl18 for M2 macrophages (Table 2) [46].

**Table 2:** Main characteristics of M1 and M2 macrophages. The table summarizes the main properties of M1 and M2 macrophages, their inducers, surface markers, and produced cytokines and chemokines.

Characteristics	M1 macrophages	M2 macrophages
Normal conditions	Inflammation	Wound healing
Tumor relation	Anti-tumorigenic, recruitment of cytotoxic T cells	Pro-tumorigenic, tissue remodeling, immunosuppression, angiogenesis, chronic inflammation
Disease prognosis	good	bad
Inducers	T_H1_ lymphocytes LPS, IFN-*γ*, IL-12, GM-CSF	T_H2_ lymphocytes IL-4, IL-6, IL-10, IL-13, CCL2, CXCL4, TGF-β, M-CSF
Markers	CD80, CD86, TLR2, TLR4, MHC II (antigen presentation)	CD206, CD163
NFκB	p65/p50	p50/p50
produced cytokines/chemokine	IL12, IL23	IL10

Under normal conditions, M2-like macrophages are activated by Th2 lymphocytes and support wound healing and tissue repair. However, the majority of cytokines in the TME supports the polarization into M2 macrophages. The M2 phenotype is especially present in the late stages of tumorigenesis. In ovarian cancer, M2 macrophages play a significant role for the progression of the disease, supporting tumor growth, metastases, angiogenesis, immunosuppression, and tissue remodeling. The presence of a high number M1 macrophages in ovarian cancer is associated with better prognosis, because of the increased recruitment of the cytotoxic T cells [46].

NFκB is a central regulator for the response of macrophages to signals from the TME and the transcription of pro- and anti-inflammatory genes. The differentiation of monocytes into macrophages and the polarization into different phenotypes occur in an NFκB-dependent manner. While the M1 macrophages express the NFκB heterodimer p65/p50, the M2-macrophages are associated with the overexpression of its p50 subunit, resulting in a p50/p50 homodimer. This homodimer prevents the transcription of pro-inflammatory genes [48, 49]. It has also been shown that the inhibition of the NFκB activator, IKKβ re-educates the M2 macrophages back to the M1 phenotype [9]. Interestingly, the long-term systemic treatment with the NFκB inhibitor thymoquinone *in vivo*, in ovarian cancer, expectedly causes the decreased growth of the primary tumor. However, these patients show a paradoxical increase of the ascitic fluid in the peritoneum, elevated infiltration of the M2 macrophages with strong NFκB activation and high concentrations of VEGF, IL-10, and CCL2. In contrast, the prolonged *in vitro* treatment of ovarian cancer cells with thymoquinone did not result in NFκB activation, suggesting that there are specific factors within the TME which support the activation of NFκB in TAMs, *in vivo* [50].

## The role of caspase-8 in macrophage differentiation

**Fig. 2:**
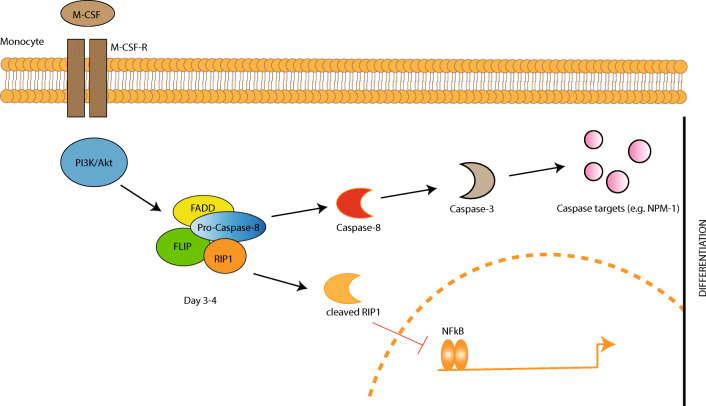
The role of caspase-8 in macrophagic differentiation. The interaction of M-CSF with its receptor provokes the PI3K/Akt pathway in the monocytes, which within 3–4 days induces the assembly of a molecular platform that includes FADD, FLIP, RIP1, and pro-caspase-8. As a consequence, the cleaved RIP1 prevents the sustained NFκB activation in the nucleus. The activated caspase-8 and -3 target downstream proteins such as nucleophosmin (NPM-1), which also regulates the transcriptional activity of NFκB. NPM-1 is a multifunctional protein, and its caspase-mediated cleavage is required for the differentiation of monocytes into macrophages after M-CSF stimulation [52, 53]

Caspase-8 has been shown to regulate the differentiation of macrophages. The significant role of caspase-8 in myeloid cells has also been observed in murine caspase-8-deficient bone marrow (BM) cells [51, 52]. After stimulation with the macrophage colony stimulating factor (M-CSF), the caspase-8-deficient BM cells were unable to differentiate into macrophages and instead underwent apoptosis. In contrast, in the presence of the granulocyte colony stimulating factor (G-CSF) or granulocyte macrophage colony stimulating factor (GM-CSF), they successfully differentiated into granulocytes and dendritic cells, respectively [51]. The treatment of human monocytes from the peripheral blood of healthy donors with M-CSF causes the oscillatory activation of the PI3K/Akt pathway, which provokes the assembly of a molecular platform including FADD, cFLIP_L_, RIPK1, and pro-caspase-8, without any interaction with the death receptors. As a result, caspase-8 is activated and cleaves RIPK1, which in turn downregulates NFκB activation, leading to the differentiation of monocytes to macrophages. In contrast, G-CSF and GM-CSF do not induce caspase-8 activation. These results suggest that caspase-8 activation is required for the differentiation of macrophages and prevention of the sustained activation of NFκB during the process. In contrast to the transient NFκB activation during macrophage differentiation, dendritic cell differentiation involves sustained NFκB activity and requires no caspase-8 cleavage [52, 53]. However, the exact molecular mechanism of caspase-8 activation in monocytes, undergoing differentiation to macrophages, remains unclear (Fig. 2).

**Fig. 3:**
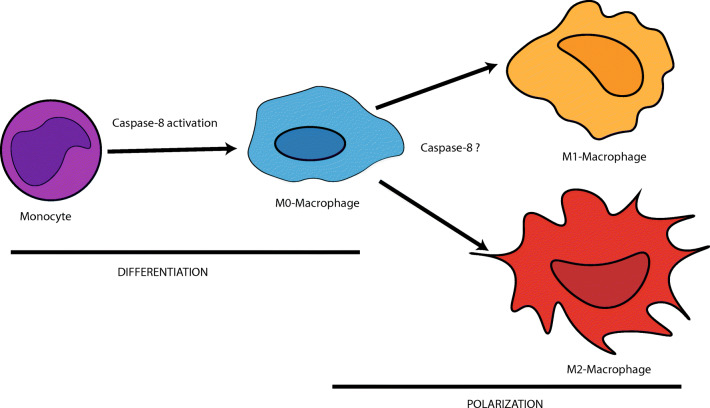
Caspase-8 is required for the differentiation of monocytes into M0 macrophages. The polarization of macrophages into M1 and M2 phenotypes seems to involve caspase-8. However, it is still unclear which molecular pathways implicate caspase-8 during the polarization and whether the process leads to up- or downregulation of caspase-8 expression

The polarization of macrophages can be manipulated with both reversible and irreversible caspase-8 inhibitors (IETD-CHO and ZIEDT-FMK respectively), causing increased autophagy and M2 polarization, mimicking the effects of two of the most prominent cytokines in the TME: CCL2 and IL-6 [54]. Both molecules intensify the expression of each other and promote the upregulation of cFLIP_L_ as well as the hyper-activation of autophagy as a cell protective mechanism. The stimulated monocytes show a significant increase of the mannose receptor CD206, a surface marker for the M2-like cells. IL-6 additionally favors the differentiation of monocytes to macrophages rather than to dendritic cells, thereby regulating antigen presentation [55]. The effect of the caspase-8 inhibitors could be reversed by the subsequent treatment with autophagy inhibitors such as leupeptin, indicating a role of caspase-8 and autophagy in macrophage polarization [54]. Cuda et al. had demonstrated in a mouse model that caspase-8 controls the polarization of macrophages, because its absence in the myeloid cells prevented the normal polarization into M1 phenotype [56]. Caspase-8 is, therefore, required not only for the elimination of old or defective blood cells but also for determining the fate of monocytes undergoing differentiation and polarization (Fig. 3).

Recently, TAMs were selectively targeted by the anti-cancer drugs trabectedin (Yondelis®) and lurbinectedin (Zepsyre®), respectively, inducing caspase-8-mediated apoptosis [57]. These drugs affect both (1) the primary tumor growth by binding to DNA, inducing double strand breaks, preventing DNA repair, and inhibiting the active transcription through structural changes of DNA and degradation of RNA polymerase II through the ubiquitin-proteasome pathway [58], and (2) the TME via modulation of the cytokine expression in the cancer cells through regulation of their transcription and depletion of TAMs by inducing overexpression of TRAIL receptors, their recruitment in lipid rafts, ligand-independent activation, and subsequent caspase-8-mediated apoptosis. Normal tissue macrophages, B, T, and NK cells remain unaffected, because they are expressing mainly decoy TRAIL receptors [57, 59]. Trabectedin and lurbinectedin also reduce the production of growth factors, angiogenic and pro-inflammatory mediators such as CCL2, IL-6, CXCL8, MIF, and IL-6 in cancer cells, monocytes, and TAMs [60, 61]. The expression of CCL2 is associated with the presence of caspase-8 in the FADDosome after TRAIL-receptor activation [62]. Caspase-8 seems to regulate angiogenesis also via the IL-1β-mediated pathway [63], which will be discussed more in detail in the next section. Thus, the downregulation of cytokine expression and angiogenesis by trabectedin and lurbinectedin could be based on a direct or indirect regulation of caspase-8, which highlights a possible therapeutic role of caspase-8 in the modulation of TME.

Trabectedin was approved for the second-line treatment of soft tissue sarcoma and relapsed, platinum-sensitive ovarian cancer in combination with pegylated liposomal doxorubicin (European Medical Agency EMA, 2007; Food and Drug Agency FDA, 2015). Lurbinectedin was approved for the treatment of soft tissue sarcoma (FDA, 2018). Recently, lurbinectedin was tested in platinum-resistant ovarian cancer against standard therapy pegylated liposomal doxorubicin and topotecan (NCT02421588). The drug missed the primary endpoint, 30% progression-free survival (PFS), and although lurbinectedin had no PFS benefit over the standard therapy, the efficacy and safety profiles of the treatments were similar, suggesting a promising role of the molecule in future therapies [64].

In summary, caspase-8 regulates the macrophage differentiation and polarization as well as the release of cytokines by tumors and immune cells. Thus, it is possible that caspase-8 could shift the balance between (i) circulating monocytes and differentiated tumor-associated macrophages, (ii) M1 and M2 macrophages, and (iii) anti- and pro-tumorigenic cytokine microenvironment.

## The role of caspase-8 in B and T cells

Caspase-8 is as an essential regulator of B and T lymphocytes. The generation of a mouse model with caspase-8 deletion specifically in the T cell lineage enabled Salmena et al. to evaluate the role of caspase-8 in adaptive immunity [65]. The normal development of thymocytes was not impaired by the absence of caspase-8, but the number of peripheral T cells was decreased significantly. The ablation of caspase-8 in this mouse model disrupted the T cell-mediated immunity and caused immunodeficiency. Murine casp8^−/−^ T cells produced low levels of IL-2, which is essential for their proliferation and activation, and were unable to respond to exocrine IL-2 stimulation. In humans, T cells with caspase-8 mutations are completely unable to produce IL-2 [65]. In contrast, caspase-8 is not required for B cell proliferation, but it is acutely necessary for B cell activation and antibody production. The absence of caspase-8 in B cells results in decreased immune response to viral and microbial infections [66].

T cells in the TME can be classified into anti- and pro-tumorigenic subgroups. Helper (CD4^+^) T cells and cytotoxic (CD8^+^) have strong anti-tumoral activity. CD8^+^ lymphocytes directly kill the cancer cells, while the CD4^+^ cells (Th1- and Th2-lymphocytes) provoke the immune response against cancer cells, producing pro-inflammatory cytokines, recruiting other immune cell and interacting with the B cells to induce antibody production. Infiltration of the T cells into tumors is associated with longer survival and better therapy response [67, 68]. However, tumors manipulate the antigen presentation producing mediator molecules such as TGF-β, IL-10, IL-6, and CCL22 and favor the switch of the T cells into regulatory FoxP3^+^ T cells (*T*_reg_). Under normal conditions *T*_reg_ cells regulate the immune tolerance, preventing the over-reactivity of immune cells. In cancers, *T*_reg_ cells suppress the immunity against the tumor cells. However, the literature about the role of *T*_reg_ cells in cancers is inconsistent. Some researchers have found a correlation between *T*_reg_ cell infiltration and poor disease prognosis [69], while others have suggested a beneficial role of the regulatory T lymphocytes in preventing cancer development suppressing chronic inflammation [70, 71]. Moreover, the role of B cells in the TME is poorly understood. The infiltration of CD19^+^ B cells correlates with poor prognosis [72], while CD20^+^ B cells have been characterized as a positive prognostic marker [73].

## Adipocytes

Ovarian cancer cells metastasize to adipose tissues, preferentially the omentum, a visceral adipose tissue connected to the organs in the peritoneal cavity, where the adipocytes provide energy sources such as fatty acids and cytokines [74]. The omentum is also rich in macrophages. Together with other immune cells they build aggregates in the adipose tissue, called “milky spots.” These are responsible for the immunity in the peritoneal cavity and support the tumor progression by producing growth and angiogenetic factors [37, 74]. Adipocytes play therefore an essential role in ovarian cancer.

Caspase-8 inhibition is linked to a reduction of inflammation and insulin resistance in adipose tissues. The adipocyte-specific knock-out of caspase-8 in a mouse model showed decreased inflammation as well and improved glucose homeostasis [75]. Thus, caspase-8 plays a pivotal role in the regulation of the inflammatory processes and insulin sensitivity in adipocytes [75–77]. The expression of TRAIL receptors is elevated in adipose tissues and the treatment of pre-adipocytes and mature adipocytes with TRAIL induced increased expression of pro-inflammatory cytokines IL-6, IL-8, and CCL2 in NFκB- and ERK1/2-dependent manner. However, the expression of IL-8 and CCL2 is not affected after treatment with zFAD.fmk, which inhibits only the enzymatic activity of caspase-8. This leads to the idea that the non-enzymatic function of caspase-8 might still play a role in the production of cytokines through the FADDosome. IL-6 and CCL20 are only partially affected by the inhibitor. Therefore, caspase-8 does not seem to have a fundamental role in the production of cytokines by adipocytes, but it is highly required for their normal differentiation and metabolic regulation [78]. Keuper et al. found that the caspase-8-mediated regulation of adipocyte metabolism, after TRAIL stimulation, through the cleavage and inactivation of peroxisome proliferator activated receptor gamma (PPAR-gammaγ) leads to the reduced expression of lipogenic genes and therefore, decreased lipogenesis and glucose uptake [79, 80].

As a regulator of the homeostasis of adipocytes, caspase-8 might be therefore a possible target for new cancer therapies. Adipocytes act not only as an energy depot in cancer, but also as a source for cytokines (also called “adipokines”), stimulating tumor cell growth, homing and metastasis. The crosstalk between cancer cells and fat tissue induces a phenotypic switch, forcing the adipocytes to produce matrix metalloproteases (e.g., MMP11) and pro-inflammatory cytokines such as IL-6, IL1β, and IL-8. IL-6 is mainly overexpressed in cancer-associated adipocytes (CAA), contributing to the invasive behavior of cancer cells [81]. Injection of a female athymic nude mouse with SKOV-3 cells showed invasion into the omentum already after 20 min. Co-culture of ovarian cancer cells with omental adipocytes induced increased cytoplasmic lipid droplet formation, leading to faster growth and proliferation [82]. In a mouse model, breast cancer cells, pre-cultured with adipocytes, prefer to metastasize to the lung instead to the breast, supporting the suggestion that adipocytes affect cell behavior during metastasis [81]. Cancer cells also induce the de-lipidation and de-differentiation of adipocytes into pre-adipocytes, which attain a fibroblast-like shape [81, 83]. Adipocytes interact with macrophages through CCL2, leading to their polarization into an M2 phenotype and enrichment in the tumors, in order to support a favorable TME. The interaction between macrophages and adipocytes results in adipose tissue inflammation, fibrosis and insulin resistance [84, 85].

## The extracellular matrix

ECM is a complex network of macromolecules, such as collagen, laminin, and proteoglycans [86]. This network regulates the cell behavior under physiological as well as pathological conditions. In the TME, the ECM is remodeled not only by the tumor cells but also by M2-macrophages, contributing to cell migration and tumor growth. It has been shown that the ECM has an impact on immune cell infiltration and angiogenesis. The generation of a neuroblastoma mouse models with caspase-8 deletion in the neural crest lineage, showed increased metastases in the bone marrow, compared to caspase-8 wild-type neuroblastoma mice [87]. Caspase-8 knockout causes the upregulation of collagen and lamin in the ECM. These changes lead to increased stiffness and fibrosis in the tissue and favor the detachment of tumor cells [87]. Under normal conditions, wound healing involves fibrosis, which is associated with the downregulation of caspase-8 [88]. This leads to the idea that the downregulation of caspase-8 in tumors such as those of ovarian or breast may be related to the onset of increased fibrosis and stiffness. Fibrosis also induces the upregulation of TGFβ, which supports T_reg_ cells, immunosuppression, and angiogenesis [67]. Integrins mediate cell adhesion to ECM. Caspase-8 interacts with them and induces the so-called integrin mediated death (IMD) of anoikis, a programmed cell death of detached “homeless” cells, located in an inappropriate microenvironment. The loss of caspase-8 and the failure of the elimination of anoikis lead to cell survival and enable cell migration and metastases [14, 89, 90].

## The interplay between caspase-8 and NFκB

It is evident that caspase-8 performs many of its functions through NFκB activation. Thus, the interplay between caspase-8 and NFκB is essential for NFκB-dependent tumors (ovarian, breast, gastrointestinal cancer, and glioblastoma). Especially interesting are the caspase-8 mutations such as G325A, D210A/D216A/G325A, which are able to activate NFκB even stronger than the wild-type protein. However, the alterations and the deletion of the caspase-8 DED domains cannot promote the activation of NFκB, because they are crucial for this signal pathway in a still unclear way [24].

Ovarian cancer subtypes with increased expression of caspase-8 are associated with higher infiltration of T cells, better response to chemotherapy, and longer overall survival (OS) [7]. These subgroups also show higher NFκB activity, indicating synergistic functions of both the molecules. Ovarian cancer cells with low caspase-8 expression are resistant to apoptosis, but when the IKKβ inhibitor IV, which suppresses the NFκB pathway, is combined with the SMAC mimetic Birinapat, which inhibits cIAP, cancer cells are forced toward necroptosis in a RIPK1-dependent manner. Overall, this demonstrates that patients with low caspase-8 levels could benefit from therapeutic strategies that rely on inducing necroptosis [7]. In contrast, caspase-8 expression increases in glioblastoma, promoting sustained NFκB activation and the transcription of several cytokines. The levels of IL-8, IL-6, IL1β, CCL2, and VEGF correlate with caspase-8 expression levels, thereby painting caspase-8 as a modulator of angiogenesis and inflammation in glioblastoma [91, 92].

As previously described, caspase-8 regulates the differentiation of BM cells by controlling the activity of NFκB. It has been shown that the crosstalk between caspase-8 and NFκB occurs in other immune cells as well. Furthermore, the stimulation of TNFR, TRAILR, TLR4, T cell receptor (TCR), and B cell receptor (BCR) activates NFκB in a caspase-8-dependent manner [93]. Lymphocyte activation through antigen receptors (e.g., TCR and BCR) is related to NFκB activation, which can be prevented after treatment with the caspase inhibitor zVAD-fmk[94]. The inhibition or the knockout of caspase-8 in lymphocytes prevent the nuclear translocation of NFκB and impairs the transcription of inflammatory genes [66, 94]. In contrast to these findings, Selmena et al. found that the absence of caspase-8 in T cells does not impair the NFκB signaling, but only affects the proliferation, activation, and cytokine responsiveness of the T cells [65]. The stimulation of BCR, TCR, and TLR4 failed to activate caspase-8-deficient B cells and Jurkat T cells compared to the wild-type cells. In contrast, caspase-8-deficient T cells could be activated through TNFR, suggesting a selective role of caspase-8 in the lymphocyte activation depending on the activated receptor. Despite the fact, that the exact molecular pathways involving caspase-8 after the stimulation of different receptors in immune cells are still unclear, the literature is consistent about the essential role of caspase-8 in the activation of NFκB and the immune response[65, 66, 93, 94].

## The regulation of the expression of cytokines and chemokines by caspase-8

Besides the variety of cells, the TME is also rich in cytokines, chemokines, and growth factors. These soluble factors modulate the cell composition and the paracrine interactions between these cells. They are produced not only by the immune cells but also by the tumor cells themselves. Caspase-8 has been found to be involved in the expression of cytokines and chemokines in different cancer cells.

TRAIL-Rs are usually associated with caspase-8-mediated apoptosis. However, they have been found to provoke a caspase-8-mediated production of pro-inflammatory cytokines as well. The TRAIL-mediated cytokine production requires the presence but not the activity of caspase-8 in the FADDosome, which consists of FADD, caspase-8, and RIPK1. The inhibition of the caspase-8 activity prevents apoptosis, but not the FADDosome-mediated cytokine release, in the presence of TRAIL, while the knockdown or complete deletion of caspase-8 blocks both the events. Catalytically inactive caspase-8 mutants (G325A, D210A/D216A/G325A) cannot restore the apoptotic pathway, but they may be even more effective in mediating the cytokine expression via the increased NFκB activation [24, 62, 95]. This may explain why some cancer types promote the expression of mutated caspase-8 and why they even benefit from TRAIL-R expression. Furthermore, this could also be one reason why targeting the TRAIL-receptor fails to promote apoptosis [96].

The main resources of TRAIL-mediated cytokines are cancer cells, which survived TRAIL-stimulation. *In vivo* experiments showed that FADD deficiency resulted in a reduced tumorigenesis and decreased immune cell infiltration, indicating a supportive role of the FADDosome-mediated secretome in tumor growth. The positive correlation between TRAIL, CCL2, and the M2-myeloid markers CD206 and CCR2 receptors indicates a possible network, which promotes the polarization of monocytes into M2 macrophages and the recruitment of tumor-supportive infiltrates. Elevated CCL2 levels have been observed in tumors such as those of ovarian [97], breast [98], and prostate [99]. High CCL2 concentrations are also associated with impaired therapy response, enhanced recruitment of TAMs, and their modeling of the surrounding tissues. The positive correlation between the TRAIL-mediated FADDosome and the tumor cytokine release was also observed in different types of cancer, e.g., lung, colorectal, pancreatic, hepatocellular, and head and neck cancer [62].

IL-1β is another pro-inflammatory cytokine, which production seems to be regulated by caspase-8. IL-1β is mainly secreted by mononuclear cells. Low levels of IL-1β promote acute inflammation and recruitment of cytotoxic T lymphocytes, whereas high levels support chronic inflammation, tumor growth, angiogenesis, invasiveness, and metastases. It has been shown that some cancer types such as invasive breast cancer, melanoma, prostate cancer, acute myeloid leukemia (AML), chronic myeloid leukemia (CML), and gastric tumors overexpress IL-1β. Under physiological conditions, the cells produce no or very low levels of IL-1β, but only during inflammation [100].

Pro-IL-1β is a precursor protein, expressed in an NFκB-dependent manner. The canonical activation pathway of pro-IL-1β involves caspase-1 as a converting enzyme. The processing of the mature IL-1β depends on the inflammasome, which consists of a NOD-like receptor NLR, the adaptor protein ASC, and caspase-1. The formation of inflammasomes is triggered by danger-associated molecular patterns (DAMPs), pathogen-associated molecular patterns (PAMPs), or some anti-cancer drugs such as doxorubicin, staurosporine, 5-fluorouracil, or gemcitabine. Caspase-8 mediates the non-canonical activation of a complex, called ripoptosome [14]. This complex consists of pro-caspase-8, FADD, RIPK1, RIPK3, and cFLIP [101]. In this case, caspase-8 directly cleaves pro-IL-1β or interacts with the NLR3 inflammasome and promotes the indirect activation of IL-1β [63, 102]. Moen et al. had observed in BM-derived cells that caspase-8 promotes the upregulation of pro-inflammatory factors such as IL1β, IL6, and CXC10 through TLR3 and TLR4. The inhibition of caspase-8 resulted in increased expression of anti-inflammatory cytokines and chemokines, indicating an essential role of caspase-8 in the modulation of inflammation [103].

Due to the variety of apoptotic and non-apoptotic functions, caspase-8 is a crucial factor in tumorigenesis, tumor progression, and therapy response. On the one hand, the presence or lack of caspase-8 in the tumor is essential for the secretion of soluble factors in the TME, which regulate the immune system, tumor growth, angiogenesis, and metastases. On the other hand, caspase-8 expression in the immune cells regulates their response to the stimulating signals from the surrounding environment.

However, the exact role of caspase-8 in the modulation of the TME in cancers is not yet fully understood. Based on our knowledge about the regulating functions of caspase-8 in the cytokine secretion by cancer and non-cancer cells, immune response, and homeostasis of immune cells, we propose, that Caspase-8 is a possible new link in the interactions between the tumor and their surrounding environment. The detailed investigation of this relationship would enable better understanding of relevant molecular mechanisms in the TME and even provide possible new therapeutic opportunities.

## Conclusion

Caspase-8 has multiple roles in cancers by modulating both the expression profile of the tumor itself and the re-organization of the TME. Whether caspase-8 regulates the inflammatory tumor milieu in favor of tumor promotion or suppression, and should be further investigated. This would provide us with more valuable information about the clinical relevance of caspase-8 and the modified factors by caspase-8, which could be used as new drug targets to reduce the tumor-supportive properties of the TME and to improve the tumor response to the classical therapies.

Gynecological tumors such as ovarian and breast cancers are associated with increased aggressiveness and invasiveness, when caspase-8 is downregulated or is absent [25]. Therefore, drug-induced caspase-8 expression is a promising therapeutic opportunity in cancers with low Caspase-8 levels. Moreover, this could be also a method to overcome chemoresistance. Furthermore, the multiple mechanisms, which tightly regulate the enzymatic activity of caspase-8 and the switch between apoptotic and non-apoptotic functions, have to be taken into account as possible targets for new therapeutic treatments. The functions of caspase-8 are tightly modulated by cFLIP proteins, XIAP, and posttranslational modifications such as phosphorylation and ubiquitination. However, the significance of these mechanisms to regulate caspase-8 in cancers has to be further investigated [91].

Caspase-8 has therefore a dual role in cancers by modulating both the expression profile in the tumor itself and the re-organization of the TME. Whether caspase-8 regulates the inflammatory tumor milieu in favor of tumor promotion or suppression, should be further investigated, in order to evaluate the clinical relevance of caspase-8 as a modulator of the TME. This would provide us more valuable information about modified factors by Caspase-8, which could be used as new drug targets to reduce the tumor-supportive properties of the TME and to improve the tumor response to the classical therapies.

## References

[1] Siegel RL, Miller KD, Jemal A (2020). Cancer statistics, 2020. CA Cancer J Clin.

[2] Ghoneum A, Afify H, Salih Z, Kelly M, Said N (2018). Role of tumor microenvironment in the pathobiology of ovarian cancer: insights and therapeutic opportunities. Cancer Medicine.

[3] Brasseur K, Gevry N, Asselin E (2017). Chemoresistance and targeted therapies in ovarian and endometrial cancers. Oncotarget.

[4] Kossai M, Leary A, Scoazec JY, Genestie C (2018). Ovarian cancer: a heterogeneous disease. Pathobiology.

[5] Chandra A, Pius C, Nabeel M, Nair M, Vishwanatha JK, Ahmad S (2019). Ovarian cancer: current status and strategies for improving therapeutic outcomes. Cancer Med.

[6] Tothill RW, Tinker AV, George J, Brown R, Fox SB, Lade S (2008). Novel molecular subtypes of serous and endometrioid ovarian cancer linked to clinical outcome. Clin Cancer Res.

[7] Hernandez L, Kim MK, Noonan AM, Sagher E, Kohlhammer H, Wright G (2015). A dual role for caspase8 and NF-kappaB interactions in regulating apoptosis and necroptosis of ovarian cancer, with correlation to patient survival. Cell Death Discov.

[8] Hansen JM, Coleman RL, Sood AK (2016). Targeting the tumour microenvironment in ovarian cancer. Eur J Cancer.

[9] Hagemann T, Wilson J, Burke F, Kulbe H, Li NFF, Pluddemann A (2006). Ovarian cancer cells polarize macrophages toward a tumor-associated phenotype. Journal of Immunology.

[10] Maelfait J, Beyaert R (2008). Non-apoptotic functions of caspase-8. Biochemical Pharmacology.

[11] Aghababazadeh M, Dorraki N, Javan FA, Fattahi AS, Gharib M, Pasdar A (2017). Downregulation of caspase 8 in a group of Iranian breast cancer patients - a pilot study. J Egypt Natl Canc Inst.

[12] McIlwain DR, Berger T, Mak TW (2013). Caspase functions in cell death and disease. Cold Spring Harbor Perspectives in Biology.

[13] Kumar S (2007). Caspase function in programmed cell death. Cell Death and Differentiation.

[14] Mandal, R., Barron, J. C., Kostova, I., Becker, S., & Strebhardt, K. (2020). Caspase-8: The double-edged sword. *Biochim Biophys Acta Rev Cancer, 188357*. 10.1016/j.bbcan.2020.188357. 10.1016/j.bbcan.2020.188357 32147543

[15] Tummers B, Green DR (2017). Caspase-8: regulating life and death. Immunol Rev.

[16] Ichim G, Tait SW (2016). A fate worse than death: apoptosis as an oncogenic process. Nat Rev Cancer.

[17] Matthess Y, Raab M, Sanhaji M, Lavrik IN, Strebhardt K (2010). Cdk1/cyclin B1 controls Fas-mediated apoptosis by regulating caspase-8 activity. [Research Support, Non-U.S. Gov't]. Mol Cell Biol.

[18] Muzio M, Stockwell BR, Stennicke HR, Salvesen GS, Dixit VM (1998). An induced proximity model for caspase-8 activation. [Research Support, Non-U.S. Gov't Research Support, U.S. Gov't, P.H.S.]. J Biol Chem.

[19] Luo X, Budihardjo I, Zou H, Slaughter C, Wang X (1998). Bid, a Bcl2 interacting protein, mediates cytochrome c release from mitochondria in response to activation of cell surface death receptors. [Research Support, Non-U.S. Gov't Research Support, U.S. Gov't, P.H.S.]. Cell.

[20] Yadav VK, Lakshmi G, Medhamurthy R (2005). Prostaglandin F2alpha-mediated activation of apoptotic signaling cascades in the corpus luteum during apoptosis: involvement of caspase-activated DNase. J Biol Chem.

[21] Bejarano I, Rodriguez AB, Pariente JA (2018). Apoptosis is a demanding selective tool during the development of fetal male germ cells. Front Cell Dev Biol.

[22] Soung YH, Lee JW, Kim SY, Jang J, Park YG, Park WS (2005). CASPASE-8 gene is inactivated by somatic mutations in gastric carcinomas. Cancer Res.

[23] Soung YH, Lee JW, Kim SY, Sung YJ, Park WS, Nam SW (2005). Caspase-8 gene is frequently inactivated by the frameshift somatic mutation 1225_1226delTG in hepatocellular carcinomas. Oncogene.

[24] Ando M, Kawazu M, Ueno T, Fukumura K, Yamato A, Soda M (2013). Cancer-associated missense mutations of caspase-8 activate nuclear factor-kappaB signaling. Cancer Sci.

[25] Stupack DG (2013). Caspase-8 as a therapeutic target in cancer. Cancer Lett.

[26] Braga Lda C, Silva LM, Ramos AP, Piedade JB, Vidigal PV, Traiman P (2014). Single CpG island methylation is not sufficient to maintain the silenced expression of CASPASE-8 apoptosis-related gene among women with epithelial ovarian cancer. Biomed Pharmacother.

[27] Kim M, Hernandez L, Annunziata CM (2016). Caspase 8 expression may determine the survival of women with ovarian cancer. Cell Death & Disease.

[28] Yan XY, Zhong XR, Yu SH, Zhang LC, Liu YN, Zhang Y (2019). p62 aggregates mediated caspase 8 activation is responsible for progression of ovarian cancer. J Cell Mol Med.

[29] Aaboud M, Aad G, Abbott B, Abdinov O, Abeloos B, Abhayasinghe DK (2019). Observation of electroweak production of a same-sign w boson pair in association with two jets in pp collisions at sqrt[s] = 13 TeV with the ATLAS Detector. Phys Rev Lett.

[30] Braga LDC, Goncales NG, Furtado RS, Andrade WP, Silva LM, Silva Filho ALD (2020). Apoptosis-related gene expression can predict the response of ovarian cancer cell lines to treatment with recombinant human TRAIL alone or combined with cisplatin. Clinics (Sao Paulo).

[31] Hensley P, Mishra M, Kyprianou N (2013). Targeting caspases in cancer therapeutics. Biol Chem.

[32] Stone ML, Chiappinelli KB, Li H, Murphy LM, Travers ME, Topper MJ (2017). Epigenetic therapy activates type I interferon signaling in murine ovarian cancer to reduce immunosuppression and tumor burden. Proc Natl Acad Sci U S A.

[33] Koschny R, Brost S, Hinz U, Sykora J, Batke EM, Singer S (2013). Cytosolic and nuclear caspase-8 have opposite impact on survival after liver resection for hepatocellular carcinoma. BMC Cancer.

[34] Manzo-Merino J, Massimi P, Lizano M, Banks L (2014). The human papillomavirus (HPV) E6 oncoproteins promotes nuclear localization of active caspase 8. Virology.

[35] Muller I, Strozyk E, Schindler S, Beissert S, Oo HZ, Sauter T (2020). Cancer cells employ nuclear caspase-8 to overcome the p53-dependent G2/M Checkpoint through Cleavage of USP28. Mol Cell.

[36] Coleman RE, Gregory W, Marshall H, Wilson C, Holen I (2013). The metastatic microenvironment of breast cancer: clinical implications. Breast.

[37] Ghoneum A, Afify H, Salih Z, Kelly M, Said N (2018). Role of tumor microenvironment in the pathobiology of ovarian cancer: insights and therapeutic opportunities. [Review]. Cancer Med.

[38] Soysal SD, Tzankov A, Muenst SE (2015). Role of the tumor microenvironment in breast cancer. Pathobiology.

[39] Hu M, Polyak K (2008). Microenvironmental regulation of cancer development. Current Opinion in Genetics & Development.

[40] Wang XP, Zhao XB, Wang K, Wu L, Duan T (2013). Interaction of monocytes/macrophages with ovarian cancer cells promotes angiogenesis in vitro. Cancer Science.

[41] Weigert A, Tzieply N, von Knethen A, Johann AM, Schmidt H, Geisslinger G (2007). Tumor cell apoptosis polarizes macrophages - role of sphingosine-1-phosphate. Molecular Biology of the Cell.

[42] Paez D, Labonte MJ, Bohanes P, Zhang W, Benhanim L, Ning Y (2012). Cancer dormancy: a model of early dissemination and late cancer recurrence. Clin Cancer Res.

[43] Ahmed N, Stenvers KL (2013). Getting to know ovarian cancer ascites: opportunities for targeted therapy-based translational research. Front Oncol.

[44] Lengyel E (2010). Ovarian cancer development and metastasis. Am J Pathol.

[45] Jiang, Y., Wang, C., & Zhou, S. (2020). Targeting tumor microenvironment in ovarian cancer: premise and promise. *Biochim Biophys Acta Rev Cancer, 188361*. 10.1016/j.bbcan.2020.188361. 10.1016/j.bbcan.2020.188361 32234508

[46] Gupta, V., Yull, F., & Khabele, D. (2018). Bipolar tumor-associated macrophages in ovarian cancer as targets for therapy. *Cancers (Basel), 10*(10). 10.3390/cancers10100366. 10.3390/cancers10100366 PMC6210537 30274280

[47] Prenen H, Mazzone M (2019). Tumor-associated macrophages: a short compendium. Cellular and Molecular Life Sciences.

[48] Mancino A, Lawrence T (2010). Nuclear Factor-kappa B and Tumor-Associated Macrophages. Clinical Cancer Research.

[49] Sica A, Saccani A, Bottazzi B, Polentarutti N, Vecchi A, van Damme J (2000). Autocrine production of IL-10 mediates defective IL-12 production and NF-kappa B activation in tumor-associated macrophages. J Immunol.

[50] Wilson AJ, Saskowski J, Barham W, Khabele D, Yull F (2015). Microenvironmental effects limit efficacy of thymoquinone treatment in a mouse model of ovarian cancer. Mol Cancer.

[51] Kang TB, Ben-Moshe T, Varfolomeev EE, Pewzner-Jung Y, Yogev N, Jurewicz A (2004). Caspase-8 serves both apoptotic and nonapoptotic roles. J Immunol.

[52] Rebe C, Cathelin S, Launay S, Filomenko R, Prevotat L, L'Ollivier C (2007). Caspase-8 prevents sustained activation of NF-kappaB in monocytes undergoing macrophagic differentiation. Blood.

[53] Guery L, Benikhlef N, Gautier T, Paul C, Jego G, Dufour E (2011). Fine-tuning nucleophosmin in macrophage differentiation and activation. Blood.

[54] Roca H, Varsos ZS, Sud S, Craig MJ, Ying C, Pienta KJ (2009). CCL2 and interleukin-6 promote survival of human CD11b + peripheral blood mononuclear cells and induce M2-type macrophage polarization. J Biol Chem.

[55] Chomarat P, Banchereau J, Davoust J, Palucka AK (2000). IL-6 switches the differentiation of monocytes from dendritic cells to macrophages. Nature Immunology.

[56] Cuda CM, Misharin AV, Khare S, Saber R, Tsai F, Archer AM (2015). Conditional deletion of caspase-8 in macrophages alters macrophage activation in a RIPK-dependent manner. Arthritis Res Ther.

[57] Belgiovine C, Bello E, Liguori M, Craparotta I, Mannarino L, Paracchini L (2017). Lurbinectedin reduces tumour-associated macrophages and the inflammatory tumour microenvironment in preclinical models. Br J Cancer.

[58] D'Incalci M, Galmarini CM (2010). A Review of Trabectedin (ET-743): A unique mechanism of action. Molecular Cancer Therapeutics.

[59] Germano G, Frapolli R, Belgiovine C, Anselmo A, Pesce S, Liguori M (2013). Role of macrophage targeting in the antitumor activity of trabectedin. Cancer Cell.

[60] Germano G, Frapolli R, Simone M, Tavecchio M, Erba E, Pesce S (2010). Antitumor and anti-inflammatory effects of trabectedin on human myxoid liposarcoma cells. Cancer Res.

[61] Larsen AK, Galmarini CM, D'Incalci M (2016). Unique features of trabectedin mechanism of action. Cancer Chemother Pharmacol.

[62] Hartwig T, Montinaro A, von Karstedt S, Sevko A, Surinova S, Chakravarthy A (2017). The TRAIL Induced Cancer Secretome Promotes a Tumor-Supportive Immune Microenvironment via CCR2. Mol Cell.

[63] Antonopoulos C, Dubyak GR (2014). Chemotherapy engages multiple pathways leading to IL-1beta production by myeloid leukocytes. Oncoimmunology.

[64] Gaillard S, A O, Ray-Coquard IL, Vergote IB, Scambia G, Colombo N, Ghamande SA, Soto-Matos A, Fernandez CM, Kahatt C, Gomez J, Nieto A, Torres N, Pardo-Burdalo B, Papai Z, Kristeleit R, O'Malley DM, Benjamin I, Pautier P, Lorusso D (2018). Phase III trial of lurbinectedin versus PLD or topotecan in platinum-resistant ovarian cancer patients: results of CORAIL trial. Annals of Oncology.

[65] Salmena L, Lemmers B, Hakem A, Matysiak-Zablocki E, Murakami K, Au PYB (2003). Essential role for caspase 8 in T-cell homeostasis and T-cell-mediated immunity. Genes & Development.

[66] Lemmers B, Salmena L, Bidere N, Su H, Matysiak-Zablocki E, Murakami K (2007). Essential role for caspase-8 in toll-like receptors and NF kappa B signaling. Journal of Biological Chemistry.

[67] Drakes, M. L., & Stiff, P. J. (2018). Regulation of ovarian cancer prognosis by immune cells in the tumor microenvironment. *Cancers (Basel), 10*(9). 10.3390/cancers10090302. 10.3390/cancers10090302 PMC6162424 30200478

[68] Santoiemma PP, Powell DJ (2015). Tumor infiltrating lymphocytes in ovarian cancer. Cancer Biol Ther.

[69] Grivennikov SI, Greten FR, Karin M (2010). Immunity, inflammation, and cancer. Cell.

[70] Savage PA, Malchow S, Leventhal DS (2013). Basic principles of tumor-associated regulatory T cell biology. Trends Immunol.

[71] deLeeuw RJ, Kost SE, Kakal JA, Nelson BH (2012). The prognostic value of FoxP3+ tumor-Infiltrating Lymphocytes in cancer: a critical review of the literature. Clinical Cancer Research.

[72] Yang C, Lee H, Jove V, Deng J, Zhang W, Liu X (2013). Prognostic significance of B-cells and pSTAT3 in patients with ovarian cancer. PLoS One.

[73] Milne K, Kobel M, Kalloger SE, Barnes RO, Gao D, Gilks CB (2009). Systematic analysis of immune infiltrates in high-grade serous ovarian cancer reveals CD20, FoxP3 and TIA-1 as positive prognostic factors. PLoS One.

[74] Meza-Perez S, Randall TD (2017). Immunological functions of the omentum. Trends Immunol.

[75] Luk CT (2017). Caspase 8 plays a pivotal role in adipose tissue inflammatory signalling and glucose homeostasis. Canadian Journal of Diabetes.

[76] Luk CT, Shi SY, Cai EP, Sivasubramaniyam T, Krishnamurthy M, Brunt JJ (2017). FAK signalling controls insulin sensitivity through regulation of adipocyte survival. Nat Commun.

[77] Gautheron J, Vucur M, Schneider AT, Severi I, Roderburg C, Roy S (2016). The necroptosis-inducing kinase RIPK3 dampens adipose tissue inflammation and glucose intolerance. Nature Communications.

[78] Zoller V, Funcke JB, Roos J, Dahlhaus M, El Hay MA, Holzmann K (2017). Trail (TNF-related apoptosis-inducing ligand) induces an inflammatory response in human adipocytes. Scientific Reports.

[79] Keuper M, Asterholm IW, Scherer PE, Westhoff MA, Moller P, Debatin KM (2013). TRAIL (TNF-related apoptosis-inducing ligand) regulates adipocyte metabolism by caspase-mediated cleavage of PPARgamma. Cell Death & Disease.

[80] Zoller V, Funcke JB, Keuper M, Abd El Hay M, Debatin KM, Wabitsch M (2016). TRAIL (TNF-related apoptosis-inducing ligand) inhibits human adipocyte differentiation via caspase-mediated downregulation of adipogenic transcription factors. Cell Death & Disease.

[81] Dirat B, Bochet L, Dabek M, Daviaud D, Dauvillier S, Majed B (2011). Cancer-associated adipocytes exhibit an activated phenotype and contribute to breast cancer invasion. Cancer Res.

[82] Nieman KM, Kenny HA, Penicka CV, Ladanyi A, Buell-Gutbrod R, Zillhardt MR (2011). Adipocytes promote ovarian cancer metastasis and provide energy for rapid tumor growth. Nat Med.

[83] Tan JX, Buache E, Chenard MP, Dali-Youcef N, Rio MC (2011). Adipocyte is a non-trivial, dynamic partner of breast cancer cells. International Journal of Developmental Biology.

[84] Correa LH, Correa R, Farinasso CM, Dourado LPD, Magalhaes KG (2017). Adipocytes and macrophages interplay in the orchestration of tumor microenvironment: new implications in cancer progression. Frontiers in Immunology.

[85] Spencer M, Yao-Borengasser A, Unal R, Rasouli N, Gurley CM, Zhu BB (2010). Adipose tissue macrophages in insulin-resistant subjects are associated with collagen VI and fibrosis and demonstrate alternative activation. American Journal of Physiology-Endocrinology and Metabolism.

[86] van der Steen SC, Raave R, Langerak S, van Houdt L, van Duijnhoven SM, van Lith SA (2017). Targeting the extracellular matrix of ovarian cancer using functionalized, drug loaded lyophilisomes. Eur J Pharm Biopharm.

[87] Teitz T, Inoue M, Valentine MB, Zhu KJ, Rehg JE, Zhao W (2013). Th-MYCN mice with caspase-8 deficiency develop advanced neuroblastoma with bone marrow metastasis. Cancer Research.

[88] Lee P, Lee DJ, Chan C, Chen SW, Ch'en I, Jamora C (2009). Dynamic expression of epidermal caspase 8 simulates a wound healing response. Nature.

[89] Stupack DG, Teitz T, Potter MD, Mikolon D, Houghton PJ, Kidd VJ (2006). Potentiation of neuroblastoma metastasis by loss of caspase-8. Nature.

[90] Teitz T, Stupack DG, Lahti JM (2006). Halting neuroblastoma metastasis by controlling integrin-mediated death. Cell Cycle.

[91] Fianco G, Contadini C, Ferri A, Cirotti C, Stagni V, Barila D (2018). Caspase-8: a novel target to overcome resistance to chemotherapy in glioblastoma. International Journal of Molecular Sciences.

[92] Fianco G, Mongiardi MP, Levi A, De Luca T, Desideri M, Trisciuoglio D (2017). Caspase-8 contributes to angiogenesis and chemotherapy resistance in glioblastoma. Elife.

[93] Lamkanfi M, Declercq W, Vanden Berghe T, Vandenabeele P (2006). Caspases leave the beaten track: caspase-mediated activation of NF-kappa B. Journal of Cell Biology.

[94] Su H, Bidere N, Zheng LX, Cubre A, Sakai K, Dale J (2005). Requirement for caspase-8 in NF-kappa B activation by antigen receptor. Science.

[95] Henry CM, Martin SJ (2017). Caspase-8 Acts in a Non-enzymatic role as a scaffold for assembly of a pro-inflammatory "FADDosome" complex upon TRAIL stimulation. Mol Cell.

[96] Lim B, Allen JE, Prabhu VV, Talekar MK, Finnberg NK, El-Deiry WS (2015). Targeting TRAIL in the treatment of cancer: new developments. Expert Opinion on Therapeutic Targets.

[97] Moisan F, Francisco EB, Brozovic A, Duran GE, Wang YC, Chaturvedi S (2014). Enhancement of paclitaxel and carboplatin therapies by CCL2 blockade in ovarian cancers. Mol Oncol.

[98] Wang J, Zhuang ZG, Xu SF, He Q, Shao YG, Ji M (2015). Expression of CCL2 is significantly different in five breast cancer genotypes and predicts patient outcome. International Journal of Clinical and Experimental Medicine.

[99] Zhang J, Patel L, Pienta KJ (2010). CC chemokine ligand 2 (CCL2) promotes prostate cancer tumorigenesis and metastasis. Cytokine & Growth Factor Reviews.

[100] Apte RN, Dotan S, Elkabets M, White MR, Reich E, Carmi Y (2006). The involvement of IL-1 in tumorigenesis, tumor invasiveness, metastasis and tumor-host interactions. Cancer and Metastasis Reviews.

[101] Safa, A. R. (2013). Roles of c-FLIP in apoptosis, necroptosis, and autophagy. *J Carcinog Mutagen, Suppl, 6*. 10.4172/2157-2518.S6-003. 10.4172/2157-2518.S6-003 PMC4219646 25379355

[102] Antonopoulos C, El Sanadi C, Kaiser WJ, Mocarski ES, Dubyak GR (2013). Proapoptotic chemotherapeutic drugs induce noncanonical processing and release of IL-1beta via caspase-8 in dendritic cells. J Immunol.

[103] Moen SH, Westhrin M, Zahoor M, Norgaard NN, Hella H, Stordal B (2016). Caspase-8 regulates the expression of pro- and anti-inflammatory cytokines in human bone marrow-derived mesenchymal stromal cells. Immun Inflamm Dis.

